# Outbreak of *Salmonella* Newport Infections Linked to Cucumbers — United States, 2014

**Published:** 2015-02-20

**Authors:** Kristina M. Angelo, Alvina Chu, Madhu Anand, Thai-An Nguyen, Lyndsay Bottichio, Matthew Wise, Ian Williams, Sharon Seelman, Rebecca Bell, Marianne Fatica, Susan Lance, Deanna Baldwin, Kyle Shannon, Hannah Lee, Eija Trees, Errol Strain, Laura Gieraltowski

**Affiliations:** 1 Epidemic Intelligence Service, CDC; 2 Division of Foodborne, Waterborne, and Environmental Diseases, National Center for Emerging and Zoonotic Infectious Diseases, CDC; 3 Maryland Department of Health and Mental Hygiene; 4 New York State Department of Health; 5 Food and Drug Administration; 6 Maryland Department of Agriculture

In August 2014, PulseNet, the national molecular subtyping network for foodborne disease surveillance, detected a multistate cluster of *Salmonella enterica* serotype Newport infections with an indistinguishable pulse-field gel electrophoresis (PFGE) pattern (*Xba*I PFGE pattern JJPX01.0061).* Outbreaks of illnesses associated with this PFGE pattern have previously been linked to consumption of tomatoes harvested from Virginia’s Eastern Shore in the Delmarva region and have not been linked to cucumbers or other produce items (1). To identify the contaminated food and find the source of the contamination, CDC, state and local health and agriculture departments and laboratories, and the Food and Drug Administration (FDA) conducted epidemiologic, traceback, and laboratory investigations. A total of 275 patients in 29 states and the District of Columbia were identified, with illness onsets occurring during May 20–September 30, 2014. Whole genome sequencing (WGS), a highly discriminating subtyping method, was used to further characterize PFGE pattern JJPX01.0061 isolates. Epidemiologic, microbiologic, and product traceback evidence suggests that cucumbers were a source of *Salmonella* Newport infections in this outbreak. The epidemiologic link to a novel outbreak vehicle suggests an environmental reservoir for *Salmonella* in the Delmarva region that should be identified and mitigated to prevent future outbreaks.

## Epidemiologic Investigation

A case was defined as infection with *Salmonella* Newport with PFGE pattern JJPX01.0061 (the outbreak strain) in a person with illness onset occurring during May 20–September 30, 2014. Initial interviews of ill persons conducted by state and local health officials found that travel to the Delmarva region during the incubation period was commonly reported. A structured, focused supplemental questionnaire was developed to collect detailed information on travel and exposure to restaurants, seafood, fruit, and produce, including tomatoes, in the 7 days before illness onset. Exposure frequencies were compared with the 2006–2007 FoodNet Population Survey, in which healthy persons reported foods consumed in the week before interview.† Information also was collected on illness subclusters, defined as two or more unrelated ill persons who reported eating at the same restaurant, attending the same event, or shopping at the same grocery store in the week before becoming ill.

**FIGURE 1:**
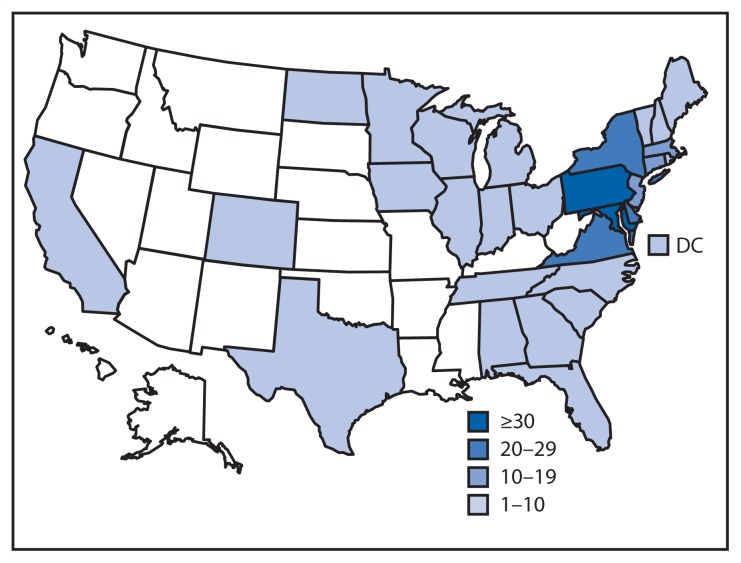
Number of persons (N = 275) infected with the outbreak strain of *Salmonella* Newport, by state — United States, May 20–September 30, 2014

**FIGURE 2:**
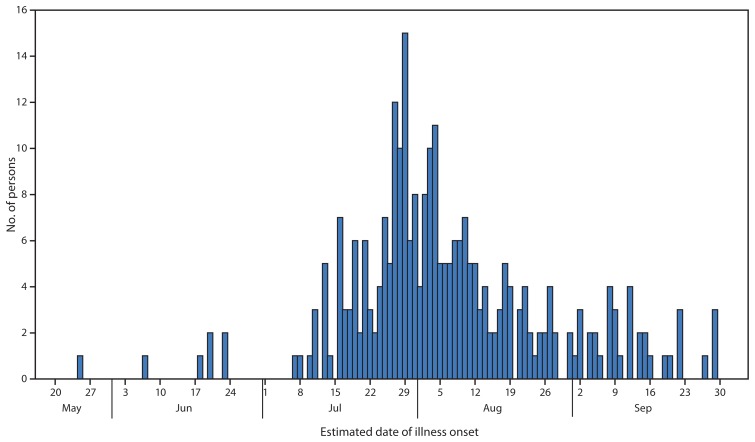
Number of persons (N = 275) infected with the outbreak strain of *Salmonella* Newport, by estimated date of illness onset — United States, May 20–September 30, 2014

A total of 275 cases were reported from 29 states and the District of Columbia (Figure 1). An additional 18 suspected cases not meeting the case definition were excluded from the analysis because they were found to be temporal outliers and unlikely to be related. Illness onset dates ranged from May 25 to September 29, 2014 (Figure 2). Median age of patients was 42 years (range = <1–90 years); 66% (174 of 265) were female. Thirty-four percent (48 of 141) were hospitalized; one death was reported in an elderly man with bacteremia. A total of 101 patients were interviewed using the supplemental questionnaire about exposures in the week before illness onset. This questionnaire focused on leafy greens and tomatoes and contained smaller sections on fruit, vegetables, and seafood common to the Delmarva region. Many patients were unreachable and did not receive the supplemental questionnaire. Sixty-two percent (49 of 79) of respondents reported eating cucumbers in the week before becoming ill. Patients were significantly more likely to report consuming cucumbers compared with respondents in the 2006–2007 FoodNet Population Survey, both for national year-round cucumber consumption (46.9% [p=0.002]) and for cucumber consumption in Maryland during the month of July (54.9% [p=0.04]). The proportion of ill persons who reported eating tomatoes, leafy greens, or any other item on the supplemental questionnaire was not significantly higher than expected compared with findings from the FoodNet Population Survey.

## Traceback investigation

Officials in Maryland, Delaware, and New York worked with their FDA district offices and FDA and U.S. Department of Agriculture foodborne outbreak rapid response teams to conduct an informational (i.e., nonregulatory) traceback from retail establishments in these states to identify a point of distribution convergence for produce items (i.e., cucumbers, leafy greens, and tomatoes) consumed in nine of 12 subclusters. Each of eight establishments in Maryland and Delaware received cucumbers from a single major distributor. Preliminary traceback from the distributor to several brokers identified a common grower on Maryland’s Eastern Shore in the Delmarva region. Traceback from a New York subcluster led to a different distribution chain than in Maryland and Delaware. Officials from the Maryland Department of Agriculture, the Maryland rapid response team, and the FDA Baltimore District Office visited the Maryland farm. Officials collected 48 environmental samples from areas where cucumbers were grown, harvested, and packed. Sediment and manure samples were taken from the farm. No samples yielded *Salmonella*; however, sampling was performed several months after the harvest. Records and interviews indicated that the farm applied poultry litter approximately 120 days before harvest, but it was not available for testing.

## Laboratory investigation

Twelve distinct illness subclusters were identified across four states, ranging in size from two to six cases. WGS was performed on 58 clinical isolates by state health departments, FDA, and CDC laboratories to further characterize the genetic relatedness of bacteria isolated from patients. Phylogenetic analysis revealed a primary group of highly related clinical isolates from cases in Delaware, Maryland, Ohio, Pennsylvania, and Virginia (median single nucleotide polymorphism distance = 26 [97.5% confidence interval = 1–37]). An additional group of highly related isolates from patients in New York was also identified, but this group was distinct from the primary phylogenetic group, consistent with the epidemiologic and traceback findings (single nucleotide polymorphism distance between the two phylogenetic groups = 102 [97.5% confidence interval = 85–114]). CDC’s National Antimicrobial Resistance Monitoring System laboratory conducted antibiotic resistance testing on three isolates from ill persons with the outbreak strain. All three were susceptible to all antibiotics tested.§

### Discussion

The epidemiologic data, traceback investigations, and whole genome sequencing all support the hypothesis that cucumbers were a likely source of *Salmonella* Newport infections in this outbreak. Cucumbers were the only food eaten by patients significantly more often than expected. Traceback investigations performed using invoices from illness subclusters in Maryland and Delaware identified a common grower of cucumbers in the Delmarva region. This is the first multistate outbreak of *Salmonella* Newport implicating a fresh produce item grown in the Delmarva region other than tomatoes. Historically, *Salmonella* Newport outbreaks associated with this PFGE pattern have been linked to red round tomatoes grown on Virginia’s Eastern Shore. These outbreaks occurred in 2002 (333 persons), 2005 (72 persons), 2006 (115 persons), and 2007 (65 persons), with an additional suspected outbreak in 2010 (51 persons) (1). A definitive contamination source has not been found, and *Salmonella* Newport has not been isolated directly from any Delmarva region tomatoes. Wildlife have been evaluated as a possible source of contamination, but fecal specimens from deer, turtles, and birds have been negative and do not support the hypothesis that animals are a source (2). Other serotypes of *Salmonella* have been linked to cucumbers; most recently an outbreak of *Salmonella* Saintpaul infections was linked to imported cucumbers from Mexico in 2013 (3).

Investigating illness subclusters can provide critical clues about the source of an outbreak. Informational traceback can support the epidemiologic investigation by quickly assessing the plausibility of one or more vehicles as the source of the outbreak. Informational traceback generally can be completed much more quickly than regulatory traceback, which requires the collection of specific types of records, such as receipts, invoices, and bills of lading, at each step of the distribution chain. In this investigation, the informational traceback quickly provided a critical clue that suggested cucumbers were a likely source in the outbreak.

Consultation with independent industry experts early in an outbreak investigation also can provide important clues to help focus the investigation on certain suspected foods. Because of the suspicion that this outbreak was caused by a novel vehicle for this *Salmonella* Newport PFGE pattern, an industry consultation was held on September 11, 2014, with three independent experts from the produce industry to obtain information regarding cucumber harvesting and distribution on the Delmarva region. The consultants provided information regarding crop production and distribution practices that also helped assess the plausibility of cucumbers as an outbreak vehicle.

What is already known on this topic?

*Salmonella* is the most common bacterial cause of foodborne disease in the United States and results in the highest number of hospitalizations and deaths among foodborne pathogens. *Salmonella* Newport has historically been a common cause of tomato-associated outbreaks in the United States. The Virginia Eastern Shore in the Delmarva region has been the site of multiple outbreaks of *Salmonella* Newport infection in recent years.

What is added by this report?

In August 2014, a multistate cluster of *Salmonella enterica* serotype Newport infections with an indistinguishable pulse-field gel electrophoresis (PFGE) pattern (*Xba*I PFGE pattern JJPX01.0061) was detected, involving 275 patients in 29 states and the District of Columbia with illness onsets occurring during May 20 and September 30. Epidemiologic, product traceback, and laboratory evidence implicated cucumbers. Whole genome sequencing, used to subtype the isolates, and the traceback investigation suggested that some, but not all, of the contaminated cucumbers were from a farm in Maryland. No *Salmonella* was isolated from environmental samples taken at the farm.

What are the implications for public health practice?

The epidemiologic link to a novel outbreak vehicle from the Delmarva region, cucumbers, suggests an environmental reservoir for *Salmonella* that might also include both the Virginia and Maryland portions of the Delmarva region. Federal, state, and local public health and regulatory authorities should focus on identifying and mitigating this potential environmental reservoir to prevent future outbreaks.

Advanced molecular detection methods, including WGS, might improve discrimination of subclusters during outbreak investigations. WGS data from the subclusters in this investigation demonstrated a phylogenetic link between clinical isolates from the eight Maryland and Delaware subclusters, in addition to differentiating these clusters from a subcluster in New York. The significance of this differentiation remains unclear at this time but might suggest that some of the illnesses in New York were not related to consumption of cucumbers from the Delmarva region. This is also supported by the informational traceback from the New York establishment, which led to a different distribution chain than those of the Maryland and Delaware establishments.

The findings in this report are subject to at least two limitations. First, no case-control study was performed because illness subclusters were small. Second, not all patients in the subclusters were systematically asked about cucumber consumption.

This outbreak supports the continued evaluation of farm practices by FDA as a part of the development of a Produce Safety Rule.¶ These evaluations include conducting a risk assessment and working with the U.S. Department of Agriculture and other stakeholders. It also includes performing research to strengthen scientific support for determining appropriate intervals between application of raw manure fertilizer and harvest. The Maryland Department of Agriculture plans additional assessments in the Delmarva region before the 2015 planting season to determine whether additional or alternative “best practices” can be implemented.

Given the typical shelf life of cucumbers is 10–14 days, cucumbers from the implicated grower are no longer available for purchase or in person’s homes. Consumers and retailers should always follow safe produce handling recommendations.** Cucumbers, like all produce, should be washed thoroughly, scrubbed with a clean produce brush before peeling or cutting, and refrigerated as soon as possible to prevent multiplication of bacteria such as *Salmonella*.

## Footnotes

* Additional information available at http://www.cdc.gov/pulsenet.

† Additional information available at http://www.cdc.gov/foodnet/studies/population-surveys.html.

§ Additional information available at http://www.cdc.gov/narms/about/index.html.

¶ Available at http://www.fda.gov/food/guidanceregulation/fsma/ucm334114.htm.
